# The development and productivity of a measure for identifying low language abilities in children aged 24–36 months

**DOI:** 10.1186/s12887-023-04079-x

**Published:** 2023-09-29

**Authors:** James Law, Jenna Charlton, Philip Wilson, Robert Rush, Vicky Gilroy, Cristina McKean

**Affiliations:** 1 School of Education Communication and Language Sciences, Newcastle University, King George VI Building, Queen Victoria Rd, Newcastle upon Tyne, Tyne and Wear, NE7 1RU UK; 2 Institute of Applied Health Sciences, University of Aberdeen, Inverness, IV2 3JH UK; 3 Finn Coral Statistical Services, 16A Denham Green Terrace, Edinburgh, EH5 3PF UK; 4 Institute of Health Visiting | c/o Royal Society for Public Health, John Snow House, 59 Mansell Street, London, E1 8AN UK

**Keywords:** Child development, Language, Language delay, Surveillance, Developmental screening, Early prevention, Public health

## Abstract

**Background:**

Accurate early identification of children with low language ability is important but existing measures generally have low sensitivity. This remains an area of concern for preventive and public health services. This study aimed to create and evaluate a measure of child language, communication and related risks which can be used by community health nurses to accurately identify children with low language aged 24–30 months.

**Methods:**

The Early Language Identification Measure (ELIM) was developed and comprised five measurement sections, each measuring different aspects of development combined into a single measure. This was tested blind against a reference standard language measure, the Preschool Language Scale-5 (PLS-5), at the universal 24–30-month health visitor review in England. The threshold for likely low language was the tenth centile or below on the PLS-5. The aim was to ascertain the performance of the five individual sections in the scale, and consider the optimum combination of sections, for predicting low language ability. Specificity, sensitivity, and positive and negative predictive values were reported for each of the five sections of the ELIM alone and in conjunction with each other. The performance for children from monolingual English-speaking families and those who spoke languages other than English were also considered separately.

**Results:**

Three hundred and seventy-six children were assessed on both the ELIM identification measure and the PLS-5 with 362 providing complete data. While each section of the ELIM predicted low language ability, the optimal combination for predicting language outcome was the parent reported vocabulary checklist coupled with the practitioner observation of the child’s communication and related behaviours. This gave a sensitivity of 0·98 with a specificity of 0·63.

**Conclusions:**

A novel measure has been developed which accurately identifies children at risk of low language, allowing clinicians to target resources efficiently and intervene early.

**Supplementary Information:**

The online version contains supplementary material available at 10.1186/s12887-023-04079-x.

## Background

Low language ability in the early years, often accompanied by neurodevelopmental disorder [1, 2], is associated with poor academic progress, peer relationship problems, school exclusion [3] and its impact extends into adulthood [4, 5]. Around 10% of children are commonly considered to be at risk of persisting low language ability [6, 7]. As for other aspects of child development there is a clear social gradient in language ability [8, 9]. With rising societal health inequalities, this gradient has been exacerbated in recent years bringing with it an even greater imperative to develop preventative interventions [9, 10]. Importantly, the gaps in language and communication abilities between socially disadvantaged children and their more advantaged peers emerge early, being detectable by 18 months of age [11]. Oral language skills may be considered a public health concern [12], meriting the development of early identification measures and preventative interventions [13]. Although language interventions focussing on parent-child interaction, and shared book reading, have proven efficacy [14], identifying which children might benefit from intervention remains a challenge.

### Identification methods

Screening for low language ability, does not yet meet accepted criteria for screening programmes [15]. Given the significant consequences extending across the lifecourse for children with low language abilities, early detection could bring significant benefits to the child and potentially to wider society. The identification of developmental problems in early childhood requires integration of both parental expectations of the child and professional assessment of the performance of the child, but also needs to take in to account a range of complex, contextually influenced social and behavioural phenomena. When compared to laboratory-based screening for inherited disorders of metabolism, for example, child development represents a highly complex, multi-faceted phenomenon which may render it less amenable to traditional notions of screening [16]. As such, developmental assessments may best be seen as the starting point, opening up a conversation with the parent about their child’s development [17], potentially leading to ongoing surveillance, rather than a definitive categorisation of risk at a single time point. Nevertheless, the literature has not supported the introduction of universal developmental or indeed language screening [15], in large part because the performance of available measures has proved inadequate. For example the ASQ Communication Scale misses ~ 1/3 children with low language abilities [18].

Here we examine an alternative approach to identification drawing on data from a recent report commissioned in the UK where there has been a programme of work focusing on low language ability as a barrier to social mobility among children living with social disadvantage [19, 20]. The study aimed to develop a a process of identification to minimise the risk of missing children with low language by maximising sensitivity, which could be implemented as part of the existing universal health visitor (HV) review at 24–30 months in England. HVs are specialist nurses, and their teams often include trained early years educators. The teams are separate from but closely linked to family doctors and paediatric services and provide families with a programme of screening, immunisation and health and development reviews, supplemented by advice around health, wellbeing and parenting. A linked intervention approach was also developed [21, 22]. The resulting Early Language Identification Measure and Intervention (ELIM-I) is a three stage process. Stage one involves completion of an identification measure which was developed to assess several areas of development and experiences relevant to language (the ELIM). Children who fall below a threshold move to stage two and three. Stage two has a continued focus on identification, covering a discussion of language background, factors related to language development including behaviour, and potential referral to other agencies. In stage three parents are invited to participate in an intervention supported by the HV, involving a shared decision-making tool and resources to promote responsive parent-child interaction. Details of the development of the full ELIM-I process are reported elsewhere [21, 22] as is comparison of the performance of one component of the stage one identification measure against the measure routinely used in England [18].

This paper focusses on the development and testing of the measure used at stage one of ELIM-I for identifying children with low language ability at the universal 24–30 month review carried out by HV teams in England: the ELIM measure.

### Aims

i. To create a measure (the Study ELIM) made up of five sections measuring different aspects of development commonly used to identify children with low language ability.
ii. To test the Study ELIM measure against an external reference standard of language development carried out blind to the original Study ELIM assessment.
iii. To select the optimum combination of the five sections to maximise sensitivity with acceptable specificity: the final ELIM measure.

## Methods

The study was commissioned by Public Health England (PHE) / Department for Education during 2019 as part of the larger programme of work previously described [21].

Five geographically and demographically disparate sites in England were identified for data collection by PHE. The target sample size across the 5 sites was 1280 aiming for 256 children per site and 128 per Income of Deprivation Affecting Children Index (IDACI) decile [23]. Data was collected by Health Visitor (HV) teams and Speech and Language Therapists (SLTs) at each site. HVs and their teams are highly trained in reliable and valid developmental screening and assessment practices. In addition, all HV team members involved in the study were trained in Study ELIM delivery prior to data collection by the research team in a 2-hour training session. SLTs are accredited professionals who are specialists in assessment and intervention of children’s speech language and communication and so are specifically qualified to use standardised psychometric language assessments.

Parents of all children who attended the universal child health review at 24–30 months were invited to participate in the study. Parents received a project information sheet and consent form along with their child health review appointment letter from the HV. Informed consent was obtained from all the study participants and their legal guardian/parents by the HV conducting the Study ELIM at the review, however the Study ELIM and Preschool Language Scale (PLS-5) were completed during separate meetings. Where consent was given the HV allocated the child a unique ID code to ensure anonymity and administered the Study ELIM. The HV returned the completed assessment to the research team in the post. Children were then invited to attend a separate speech and language therapy assessment irrespective of whether they were considered to have low language abilities or not. At this appointment the reference measure, PLS-5 [24], was administered by a SLT who was blind to the outcome of the ELIM assessment. The SLT returned completed PLS-5 forms to the research team in the post. We then investigated the performance of each of the five sections of the Study ELIM with regard to predicting a child falling at the 10th centile or below on the reference standard measure, that is having low language.

**Fig. 1:**
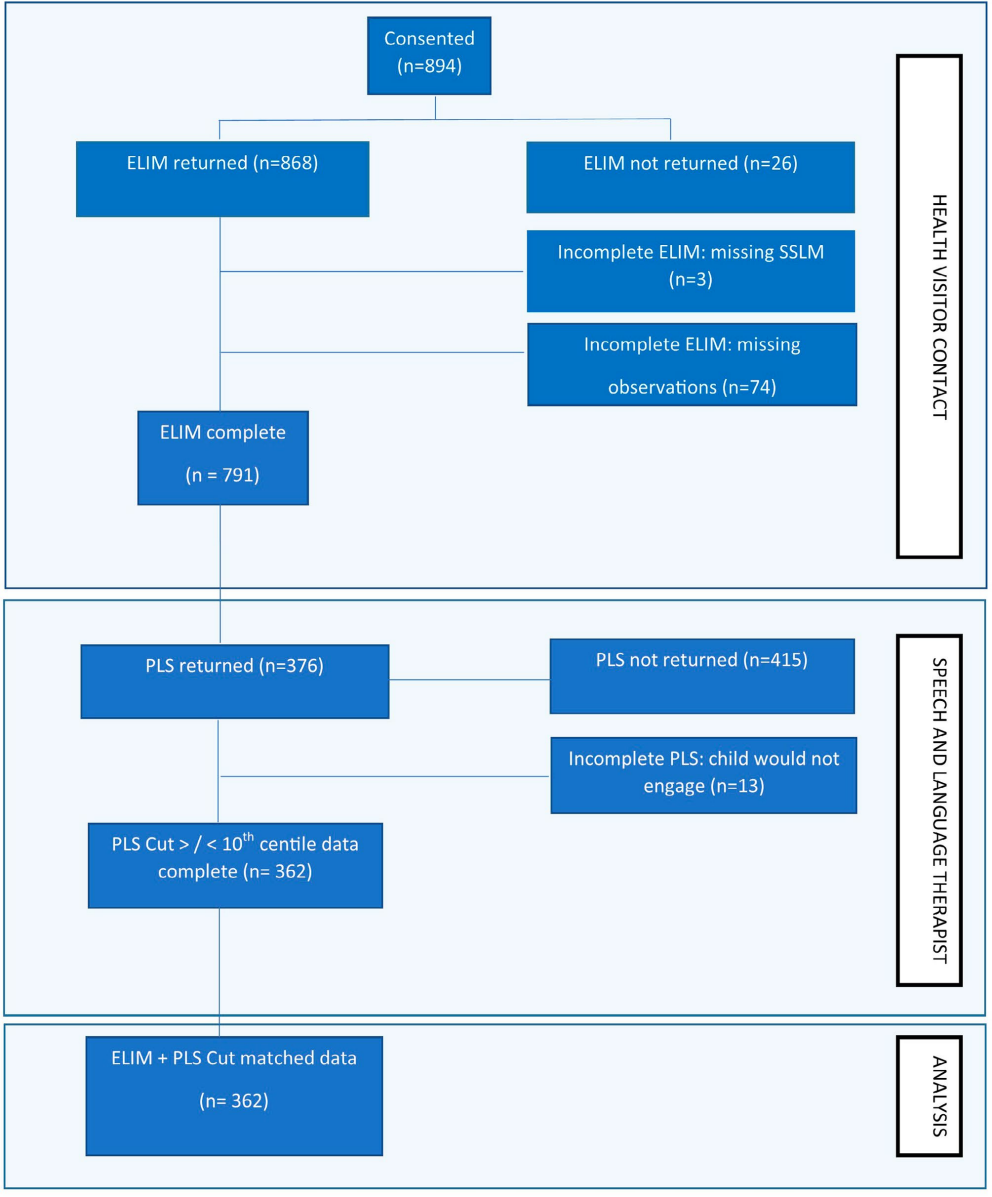
Stages of study methodology and participant flow

### Measures

#### The study early language identification measure

The Study ELIM was developed to include five sections comprising candidate items for the final measure. The sections included items commonly identified in the literature as potential indicators of early language development: (1) developmental milestones [25]; (2) a vocabulary checklist [26]; (3) family and demographic risk factors including family history, socio-economic status and parent-child interaction [27]; (4) observations made by the professionals involved in the review relating to the child’s attention, turn-taking, intelligibility, comprehension, gesture use and intentional communication [28]; and (5) parental concern [29] [see Additional file 1].

All the candidate items within the five sections were constructed by the research team drawing on relevant literature and expert knowledge in collaboration with a range of professionals (SLTs, HVs etc.) and parents using Patient and Public Involvement (PPI) processes [21]. The only exception was the vocabulary checklist section. This 50 word list, known as the Sure Start Language Measure (SSLM) [26] was originally distilled from the Communicative Development Inventory (CDI) [30]. The SSLM is standardised and was used within the UK Sure Start program, and subsequently evaluated in a number of other projects. Parents are asked to indicate whether or not their child uses the word listed. Where the child speaks and/or hears languages other than English they are credited with knowing the word if they use it or its equivalent in any of the languages spoken in their home.

The performance of these different sections in the identification of children at risk of low language (developmental milestones, vocabulary checklist, family and demographic risks, professional observation, parental concern) have never been compared in combination against a reference standard measure.

#### The reference standard

The reference standard was the PLS-5 UK version [24] which is a structured assessment of receptive and expressive language with items ranging from preverbal, interaction-based skills to emerging language. The assessment is detailed and requires practitioners to observe the child and, where applicable in terms of developmental stage, complete play-based tasks. The person administering the task must have the relevant professional accreditation. In most cases, and in the case of this study, this is a SLT. Administration typically takes 30–45 min.

Where families spoke language(s) other than English (LOTE) at home either exclusively or in addition to English, the SLTs administering the PLS-5 followed local protocols to involve interpreters as appropriate. For the purposes of analysis and in accordance with the prevalence literature the threshold on the PLS-5 total language score was set at the 10th centile or below.

#### Development of the final ELIM measure

The screening performance of the five sections of the Study ELIM, and the various combinations of these, in relation to the PLS-5 threshold, were examined and the combination with the optimal sensitivity and specificity chosen to create the final ELIM measure for clinical use.

## Results

### The sample

Three hundred and seventy six children were assessed on both the Study ELIM measure and the PLS-5 (see Fig. 1 for participant flow). The sample was slightly skewed towards lower socioeconomic status (Table 1) but included families from all socio-economic deciles as defined using the Income of Deprivation Affecting Children Index (IDACI) [23].

Two hundred and seventy-three (75%) had typical language, achieving scores above the published 10th centile threshold, while 89 (25%) had low language, here defined as scores at or below the threshold. This proportion (25%) was higher than anticipated but may reflect the characteristics of the five sites which were relatively socially disadvantaged. This proportion was similar to other studies of 2-year-old children’s language in community-ascertained samples [18, 31]. Notably the Early Language in Victoria Study which also recruited via community maternal health nurses found 19.7% of children at 2 years of age fell below the 10th centile cut-point using the Communication Development Inventories [32], perhaps suggesting the samples are more representative of the population than those used to norm the tests. There were differences between the typical language and low language groups (Table 1), with the low language group showing higher levels of disadvantage, male sex and younger age, although birthweight and gestation did not differ. Thus the PLS-5 threshold identified a low language group which was more socially disadvantaged and a little younger despite centile scores adjusting for age. The groups also differed in terms of the language spoken at home (Table 1). When families spoke LOTE at home, either exclusively or in combination with English they were less likely to attend the second appointment with SLT (X^2^ (1,672) = 19.357, *p* < .001).

Whilst UK data regarding the proportion of 2–3 year olds who are in families who speak LOTE is not available for comparison, the final proportion of children from LOTE backgrounds in our sample for analysis does reflect that of the UK school population of ~ 20% suggesting that our sample is likely representative of the wider population [33].

Of the 791 children who completed and returned the Study ELIM assessment forms, 362 also had a complete PLS-5 assessment with the SLT. Whilst families with ELIM only were broadly comparable to those with ELIM plus PLS-5 [20] there were some differences [20]. Those with only ELIM assessments were more disadvantaged (U = 57500.5, *p* < .001) and were less likely to have a family history of learning difficulties (*X*^*2*^ (1,780) = 7.193, *p* = .009).

**Table 1:** Core data by PLS-5 centile threshold

		Typical language	Low language		T test, Mann Whitney or Chi squared tests
		> 10th centile	<=10th centile	Full sample	
		N = 273	N = 89	N = 362	
Birthweight (kg)					
	Missing, N (%)	5 (1·83)	4 (4·49)	9 (2·5)	
	Mean (SD)	3·39 (0·54)	3·27 (0·85)	3·37 (0·63)	*t* (351) = 1.28, *p* = .204
Length of pregnancy (weeks)					
	Missing, N (%)	8 (2·93)	1 (1·12)	9 (2·5)	
	Mean (SD) ^1^	39·11 (1·62)	38·63 (2·13)	38·99 (1·77)	*t* (351) = 1.94, *p* = .055
Age at PLS-5 assessment (months)***					
	Missing, N (%)	13 (4·76)	1 (1·12)	14 (3·9)	
	Mean (SD)	26·09 (1·46)	25·00 (1·63)	25·82 (1·58)	*t* (346) = 5.89, *p* < .0.001
Age at ELIM assessment (months)					
	Missing, N (%)	24 (8·79)	9 (10·11)	33 (9·1)	
	Mean (SD)	19·79 (3·33)	19·71 (4·33)	19·77 (3·59)	*t* (327) = 0.14, *p* = .89
IDACI Decile ***	Missing, N (%)	10 (3·66)	8 (8·99)	18 (5·0)	
	Median (IQR)^3^	5·00 (4·0)	3·00 (2·50)	5·00 (4·00)	*U* = 7628, *p* < .001
Sex**					
	Missing, N (%)	4 (1·4)	0 (0)	4 (1·1)	
Female	N (%)	134 (49·8)	30 (33·7)	164 (45·8)	
Male	N (%)	135 (50·2)	59 (66·3)	194 (54·2)	*X*^*2*^ (1,358) = 6.99, *p* = .01
Languages spoken in the home					
	Missing, N (%)	34 (12·4)	12 (13·5)	46 (12·7)	
English only	N (%)	205 (85·8)	46 (59·7)	251 (79·4)	
Primary language other than English	N (%)	15 (6·3)	17 (22·1)	32 (10·1	
Use English and another language equally	N (%)	19 (7·9)	14 (18·2)	33 (10·4)	*X*^*2*^ (2,316) = 25.17, *p* < .001

*Key*: 1 Standard deviation; 2 IDACI Income Deprivation Affecting Children Index (UK); 3 Interquartile range

#### Screening performance of the five study ELIM sections against the PLS-5 reference standard

To investigate the screening performance of the five sections of the Study ELIM, and the various combinations of these, in relation to the PLS-5 threshold, the sections were conservatively dichotomised. Sections 1, 3, 4, and 5 (developmental milestones, family and demographic risks, professional observation and parental concern respectively) were dichotomised according to whether *any* item in the respective section was endorsed, indicating a potential issue, or *no* items were endorsed, indicating no issue. Thus no individual milestone, family or demographic factor, behaviour or concern was prioritised within sections. Where data were missing for any item(s), that section score was denoted as missing if no other item was endorsed in that section but included if at least one item in the section was selected. This scoring approach was not possible for Sect. 2, the vocabulary list, which generated a continuous score: the Receiver Operating Characteristic (ROC) curve in relation to the PLS-5 threshold generated a threshold of 18 words or above as our vocabulary threshold [18]. Table 2 presents the productivity for each section in isolation and in combination in terms of specificity, sensitivity and positive and negative predictive values. Sensitivity refers to the proportion of children correctly identified as having low language by the ELIM measure when compared to the reference measure, the PLS 5. Specificity refers to the proportion of children correctly identified as having typical language when compared to the reference measure. The positive predictive value (PPV) is the probability that children identified with low language on the ELIM measures do have low language. The negative predictive value (NPV) is the probability that children identified with typical language on the ELIM do have typical language. PPV and NPV are the measures that are most commonly used by practitioners to predict the prescence or absence of a condition and are population specific. Positive and negative likelihood ratios are also presented here for completeness (PLR, NLR). PLR represents the change in odds of a child having low language when the child is identified with low language on the ELIM. NLR represents the change in odds of a child having low language when they are identified as having typical language on the ELIM. (Formulae for calculating sensitivity, specificity, PLR, NLR, NPV and PPV are presented in Additional file 2).

When considering the individual sections and their combinations, the vocabulary checklist (Sect. 2) and the professional observation (Sect. 4) combined provided the optimal sensitivity (0.98) and specificity (0.63). The least discriminating section (Sect. 3) includes familial and social risk variables. The vocabulary checklist was most accurate in determining which children did not have a problem, defined as a language score at or below the 10th centile, than in identifying those that did. The PPV for Sects. 2 and 4 in combination is low (0.23), reflecting the relatively low prevalence of the condition in this community sample. For conditions where the consequences of not intervening are high and within systems where relatively low cost and low burden preventative interventions are available then this level of PPV is acceptable [34]6. By contrast the NPV is very high suggesting very few false negatives (> 0.99).

When we look at data where children had both the Study ELIM and the reference standard PLS-5 assessment these include 282 (79.4%) from monolingual English families, and 73 (20.6%) from families where LOTE are spoken. Additional files 3 and 4 present the productivity figures separately for children in families speaking LOTE and those from monolingual English-speaking homes. Results are broadly comparable to the sample as a whole (Monolingual English Sensitivity = 0.96 Specificity = 0.64; LOTE Sensitivity = 1.00 Specificity = 0.58).

**Table 2:** Productivity figures for each section by the language reference standard and each section in combination

	Sensitivity	Specificity	PLR	NLR	PPV	NPV
Section 1 Developmental milestones	0·88	0·55	1·93	0·23	0·18	0·98
Section 2 Word list	0·85	0·84	5·18	0·17	0·37	0·98
Section 3 Population risk factors	0·7	0·59	1·70	0·51	0·16	0·95
Section 4 Professional observations	0·91	0·65	2·62	0·14	0·23	0·98
Section 5 Parental concerns	0·73	0·74	2·85	0·36	0·24	0·96
*Combinations of 2 sections*						
Sections 1 & 2	0·93	0·54	2·02	0·13	0·18	0·99
Sections 1 & 3	0·94	0·36	1·46	0·16	0·14	0·98
Sections 1 & 4	0·97	0·44	1·71	0·08	0·16	0·99
Sections 1 & 5	0·88	0·49	1·73	0·25	0·16	0·97
Sections 2 & 3	0·96	0·52	1·99	0·09	0·18	0·99
**Sections 2 & 4**	**0·98**	**0·63**	**2·62**	**0·04**	**0·23**	**> 0·99**
Sections 2 & 5	0·89	0·7	2·96	0·16	0·25	0·98
Sections 3 & 4	0·96	0·4	1·58	0·11	0·15	0·99
Sections 3 & 5	0·89	0·47	1·66	0·24	0·16	0·97
Sections 4 & 5	0·93	0·56	2·14	0·12	0·19	0·99
*Combinations of 3 sections*						
Sections 1,2 & 3	0·97	0·35	1·49	0·10	0·14	0·99
Sections 1,2 & 4	0·99	0·43	1·74	0·03	0·16	> 0·99
Sections 1,2 & 5	0·93	0·49	1·83	0·14	0·17	0·98
Sections 1,3 & 4	0·98	0·29	1·37	0·08	0·13	0·99
Sections 1,3 & 5	0·94	0·33	1·41	0·17	0·14	0·98
Sections 2,3 & 4	0·99	0·38	1·61	0·03	0·15	> 0·99
Sections 2,3 & 5	0·96	0·44	1·70	0·10	0·16	0·99
Sections 2,4 & 5	0·98	0·55	2·19	0·04	0·2	> 0·99
Sections 3,4 & 5	0·97	0·36	1·51	0·09	0·14	0·99
*Combinations of 4 sections*						
Sections 1,2,3 & 4	0·99	0·28	1·38	0·04	0·13	> 0·99
Sections 1,2,3 & 5	0·97	0·33	1·43	0·10	0·14	0·99
Sections 1,2,4 & 5	0·99	0·4	1·64	0·03	0·15	1·00
Sections 1,3,4 & 5	0·98	0·27	1·33	0·08	0·13	0·99
Sections 2,3,4 & 5	0·99	0·35	1·52	0·03	0·14	> 0·99

*Key*: PLR = Positive Likelihood Ratio; NLR = Negative Likelihood Ratio; PPV = Positive Predictive Value; NPV = Negative Predictive Value; See Additional file 2 for definitions of sensitivity, specificity, PLR, NLR, NPV and PPV and formulae for their calculation

## Discussion

This study aimed to create and evaluate a measure of child language, communication and related risks which can be used by community health nurses to accurately identify children with low language aged 24–30 months. The goal was was to identify the optimum combination of items commonly identified in the literature as potential indicators of early language development to maximise sensitivity and with acceptable specificity. A short vocabulary checklist plus clinician observation provided excellent sensitivity and acceptable specificity for the identification of low language ability, that is language ability at or below the 10th centile, in three hundred and sixty two 24-30-month-old children. The sensitivity calculation shows that 98% of children with low language on the reference measure would be correctly identified as having low language by the final ELIM measure. The specificity calculation shows that 63% of children with typical language on the reference measure would be correctly identified as having typical language. Very few children with low language, would therefore be ‘missed’ using this approach.

A number of characteristics of the final ELIM could explain the relative sensitivity of this measure when compared to those in the wider screening literature [15]. First the short vocabulary list, supported by the practitioner, is relatively easy for families to engage with when compared to longer, independently completed vocabulary checklists often used, perhaps increasing reliability in reporting [26]. Second the inclusion of professional observation allows consideration of a number of key potential ‘red flags’ relating to attention, turn-taking, intelligibility, comprehension, gesture use and intentional communication which the research literature suggests are related to prognosis and severity [21]. Finally, the inclusion of the professional observation element invites the skilled and knowledgeable HV practitioners to bring their clinical experience to bear on the decision-making process [35].

The positive and negative predictive values (PPV and NPV) evaluate the probability that once a child has a positive or negative result that that result is correct. The NPV of > 0.99 indicates near perfect prediction that a child identified by the ELIM as having typical language will have typical language. The PPV of 0.23 suggests that we can be less sure that a child identified by the ELIM as having low language really does have low language. This relatively low positive predictive value could therefore risk over-referral to specialist services and perhaps create unnecessary worry and stigma for some families but this is a consideration in all screening programmes. We propose that such risks of negative consequences can be mitigated if an early identification tool is embedded within a process and a system where a nuanced and tailored response is offered in response to identified risk of low language [20]. We suggest that the outcome of the new final ELIM measure [36] should trigger a wider conversation with a family to understand the broader developmental profile of the child, their concerns, the child’s functional communication and developmental history and the barriers and enablers in place for the family to be able to support their child’s language development optimally [20, 21]. Taking all of this into account, and with knowledge of the available specialist services in a particular context, the HV decides whether onward referral is required. The HV also provides resources to support parents to engage in responsive interaction with their child more often during everyday activities [22]. This three stage ELIM-I approach is effectively a form of triage as suggested by Sim and colleagues [2], facilitated by shared decision-making tools, goal setting, review and tailored support. Intervention begins immediately for those requiring and waiting for specialist services; supports the development of those with milder, isolated difficulties; and, through monitoring of progress, differentiates those with persisting versus transient needs. Our PPI and qualitative work indicated that parents were keen to have such a conversation to share any concerns that they may have and that anxiety can be alleviated through the adoption of specific communication approaches [21]. Potential stigma can be addressed, at least in part, by the universal provision of the ELIM-I pathway and child language development advice being given to all families. The additional value of this approach is that it potentially leads to more equitable access to services where all parents are given the opportunity to discuss their child’s needs, needs of which they may not be aware.

For a condition such as persisting low language which has significant consequences for the individual and wider society then we posit that the level of PPV found here is acceptable [34]. In the UK, where this screening and intervention approach was designed, the psychological and economic costs of intervention are relatively low, there are services already available to manage the families’ needs, and signpost them to resources, and there is an intervention for use by practitioners to accompany the ELIM assessment. Application to other contexts would need to consider the nature of the universal early intervention platform upon which the ELIM-I would be supported. Whether such an identification process “works”, depends on the engagement of parents and the interventions that follow and further research is needed to address this issue. In essence we suggest that overly simplistic notions of ‘pass’ and ‘fail’ may not be suitable for developmental screening procedures. A more nuanced consideration of the child and family context and delivery of a range of interventions tailored to those characteristics may be more appropriate.

### Study limitations

The sites were selected because they had well developed data collection systems and with children from a representative range of different socio-demographic backgrounds. We asked HV services to engage all parents attending the universally offered 24–30 month review and, as indicated above, our sample was representative of England as a whole in terms of socio-demographic characteristics, but there is always the possibility that those less motivated to attend the review may differentially affect the outcome. In contrast, those who *did* attend the second assessment by the SLTs may have had higher levels of language difficulty, with parental concern perhaps increasing the probability of attendance.

The issue of the relevance of the reference standard to children from families speaking LOTE is more of a challenge given that no single measure could be available given the range of languages which would need to be covered. Given our aim to obtain a sample representative of the English population we chose to include children from LOTE families and use the PLS-5 with the use of interpretors. Issues of validity of the PLS-5 cutpoint for this subgroup remain and further empirical work is recommended.

It is important to note that we are advocating that the final ELIM ‘credits’ children’s knowledge in any and all languages they hear and that the measure is used in concert with a detailed conversation with the clinician. Furthermore, the observation section of the final ELIM warrants further attention because it is possible that practitioners may respond differently to childcare practices of non-western families. In the case of families speaking LOTE we therefore suggest that greater care and contextualisation is required when interpreting the final ELIM measure together with cultural awareness and sensitivity from the practitioner.

### Further research

The validity and productivity of the final ELIM measure, comprising the vocabulary checklist and the observation needs to be further tested in clinical practice together with its acceptability to parents. Initial indications suggest they have found the process useful. Ultimately the effectiveness of the process of identifying children at this early age can only be assessed in terms of better child and parental outcomes over time. This is especially important in the field of child language where patterns of development can vary considerably in the early years and this may be particularly relevant for children from LOTE backgrounds.

## Conclusion

Early identification of low language ability in young children has remained a public health concern for many years, severely limiting the potential for the provision of early preventative interventions. In this study we describe a novel measure that accurately identifies children at risk of persisting low language involving a vocabulary checklist and practitioner observation, which allows clinicians to engage with parents to discuss the nature of their child’s needs, target resources efficiently to address them, and provide intervention tailored to the child’s and family’s resources.

## Electronic supplementary material

Below is the link to the electronic supplementary material.

Supplementary Material 1

Supplementary Material 2

Supplementary Material 3

Supplementary Material 4

## Data Availability

Data have been made available to reviewers. For other purposes the data are not available. As a clinical dataset containing clinical data, we have an ethical and legal responsibility to respect participants’ rights to privacy and to protect their identity. We do not have informed consent for publication of the dataset. All data collection materials, study protocol and funding application are available from the corresponding author on request.
