# Supplementary material for: The development and productivity of a measure for identifying low language abilities in children aged 24–36 months
Source: BMC Pediatr. 2023 Sep 29;23:495. doi: 10.1186/s12887-023-04079-x (PMC10540411; doi:10.1186/s12887-023-04079-x)
Supplement: Supplementary file 1 — Supplementary Material 1 [file 12887_2023_4079_MOESM1_ESM.docx]

Additional file 1: The Study ELIM

# SECTION 1: Can you tell me about your child’s communication?

Has your child demonstrated the following things.

| **Number** | **Over the last few months** | **Yes** | **Not sure** | **No** |
| --- | --- | --- | --- | --- |
| 1 | By the time they were eighteen months was your child walking independently? |  |  |  |
| 2 | By the time they were 24 months was your child able to put two words together (mummy sock, my drink, eat dinner)? |  |  |  |
|  | **Now** | **Yes** | **Sometimes** | **No** |
| 3 | Does your child understand what people say to them? |  |  |  |
| 4 | Is your child able to find two objects when you ask them (e.g. Show me the teddy and the ball)? |  |  |  |
| 5 | Does your child ask simple questions (“Where ball?” “What Daddy doing?” “What colour?”)? |  |  |  |
| 6 | Can you understand what he/she is saying? |  |  |  |
| 7 | Can other people understand what he/she is saying? |  |  |  |
| 8 | Is your child able to talk about something they are interested in? |  |  |  |

# SECTION 2: Which words does your child say?

Please take a look at the words below and tick which words you have heard your child say.

| 1. Mummy/mum |  | 11. Aeroplane |  | 21. Towel |  | 31. Fit |  | 41. Wet |  |
| --- | --- | --- | --- | --- | --- | --- | --- | --- | --- |
| 2. Bye/bye bye |  | 12. Car |  | 22. Bed |  | 32. Like |  | 42. After |  |
| 3. No |  | 13. Book |  | 23. Settee/sofa |  | 33. Rip/tear |  | 43. Day |  |
| 4. Ball |  | 14. Milk |  | 24. School |  | 34. Shake |  | 44. This |  |
| 5. Juice |  | 15. Hat |  | 25. Friend |  | 35. Think |  | 45. Our |  |
| 6. Owch/ow |  | 16. Shoe |  | 26. Person |  | 36. Gentle |  | 46. Where |  |
| 7. Cat |  | 17. Leg |  | 27. Hello/hi |  | 37. Fast |  | 47. All |  |
| 8. Thank you |  | 18. Pillow |  | 28. Shopping |  | 38. Happy |  | 48. Much |  |
| 9. Cold |  | 19. Rubbish |  | 29. Carry |  | 39. Last |  | 49. Need to |  |
| 10. Hug/cuddle |  | 20. Plate |  | 30. Finish |  | 40. Tiny |  | 50. If |  |
| **Column Total** |  | **Column Total** |  | **Column Total** |  | **Column Total** |  | **Column Total** |  |

9: Total number of words:

# SECTION 3: Can you tell me about you and your family?

| **Number** | **Parent-child interactions** | | | | | | | | | |
| --- | --- | --- | --- | --- | --- | --- | --- | --- | --- | --- |
| 10 | Some people share books with their child although the amount of time spent doing this can vary a lot. In an average week, how often do you share books with your child? (please highlight or circle) | | | | Everyday | | 3 or 4 times | 1 or 2 times | | Too busy |
| 11 | Which activities outside the home have you enjoyed with your child this week? For example, going to the park, to the shops (please state how many) | | | |  | | | | | |
| 12 | How often do you talk to your child about the toy they are playing with ? (please highlight or circle) | | | | All the time | | Sometimes | | Very rarely | |
|  | **Family history** | | | | | | | | | |
| 13 | Does anyone in your family have a learning difficulty or a speech and language difficulty? (please highlight or circle) | | | | Yes | | No | Don’t know | | |
| 14 | If so, which of the child’s relatives has the difficulty (please highlight or circle) | Brother/sister | Mum/Dad | Aunt/Uncle | Grandparent | | | Other (please specify) | | |
| 15 | If so, please indicate what the difficulty was called (please highlight or circle) | Speech and/or language difficulties | Autism spectrum disorder (ASD) | Attention deficit hyperactivity  disorder (ADHD)? | Reading and/or writing | | | Other (please specify) | | |
| 16 | Is there any history of mental health difficulties in the family home (for example anxiety or depression)? (please highlight or circle) | | | | Yes | | No | Don’t know | | |
| 17 | How old were you (or the primary carer) when you left full time education? | | | |  | | | | | |
| 18 | What was your highest level of qualification achieved? (please highlight or circle) | | | | GCSE | Practical qual. (e.g.  NVQ) | | A-level | | Degree |
|  | **The child** | | | | | | | | | |
| 19 | Does your child suffer from any long-term health concerns requiring regular visits to the nurse or doctor? (please highlight or circle) | | | | Yes | | No | Don’t know | | |
| 20 | Has your child had recurring ear infections? (please highlight or circle) | | | | Yes | | No | Don’t know | | |

**SECTION 4: Assessors observation of the child**

Please tick which of the following behaviours you (the health professional) observed or heard when speaking with his/her carer during the 2- 2 ½ year review.

| Number |  | | | **Yes** | | **No** |
| --- | --- | --- | --- | --- | --- | --- |
| 21 | Observed communicative intent (child means to communicate something verbally to parent/carer) | | |  | |  |
| 22 | Speech mostly intelligible to parents/carers | | |  | |  |
| 23 | Observed using single words only | | |  | |  |
| 24 | Observed putting words together | | |  | |  |
| 25 | Does the child use gestures instead of spoken language to get their message across? | | |  | |  |
| 26 | Do the parent/carer and child take turns when communicating? | | |  | |  |
| 27 | Does the child understand what is being said to him/her when their parent/carer asks them something which is obvious from the context? (i.e. when showing toys to the child) | | |  | |  |
| 28 | Attention: (please circle) | Fleeting  (flits from one thing to another) | Single channelled (attention can’t easily be shifted) | | Accepts adult direction | |

# SECTION 5: Do you have any concerns about your child’s development?

| **Number** |  | **Yes** | **Sometimes** | **No** |
| --- | --- | --- | --- | --- |
| 29 | Do you have any worries/concerns about how clearly your child speaks compared to other children of the same age? |  |  |  |
| 30 | Do you have any worries/concerns about how your child uses words or speaks in short sentences compared to other children of the same age? |  |  |  |
| 31 | Do you have any worries/concerns about whether your child understands what you say to him/her compared to other children of the same age? |  |  |  |
| 32 | Physical movement and language development can sometimes be connected. Do you have any worries/concerns about how your child uses their arms and legs compared to other children of the same age? |  |  |  |
| 33 | Are you worried / concerned about your child’s behaviour compared to that of other children of the same age? |  |  |  |
