# Supplementary material for: The development and productivity of a measure for identifying low language abilities in children aged 24–36 months
Source: BMC Pediatr. 2023 Sep 29;23:495. doi: 10.1186/s12887-023-04079-x (PMC10540411; doi:10.1186/s12887-023-04079-x)
Supplement: Supplementary file 2 — Supplementary Material 2 [file 12887_2023_4079_MOESM2_ESM.docx]

Additional file 2: Definitions and formulae for productivity metrics

*Definitions*

- Sensitivity: the proportion of people WITH a given condition that have a positive result on a screening test. A test that is 100% sensitive means all individuals with a given condition are correctly identified i.e. there are no false negatives.
- Specificity: the proportion of people WITHOUT a given condition that have a negative result on a screening test. A test that is 100% specific means al individuals who do not have a given condition are correctly identified i.e. there are no false positives.
- Positive predictive value: the proportion of people with a positive result on a given screen who DO have the condition.
- Negative predictive value: the proportion of people with a negative result on a given screen who DO NOT have the condition
- Positive likelihood ratio: The change in odds of an individual having a given condition when they have a positive result on a given screen
- Negative likelihood ratio: The change in odds of an individual having a given condition when they have a negative result on a given screen

*Formulae*

|  |  | Status of individual on reference test | |
| --- | --- | --- | --- |
|  |  | Has the condition | Does not have the condition |
| Results from ‘screening’ test | Positive* | 1. True Positive | 1. False negative |
|  | Negative | 1. False Negative | 1. True Negative |

*Positive means a positive indication of the presence of a given condition.

Sensitivity=[a/(a+c)]×100

Specificity=[d/(b+d)]×100

Positive Predictive Value (PPV)=[a/(a+b)]×100

Negative Predictive Value (NPV)=[d/(c+d)]×100.

Positive Likelihood Ratio (PLR)=sensitivity/(100 – specificity)

Negative Likelihood Ratio (NLR)=(100 – sensitivity)/specificity
