# Supplementary material for: The development and productivity of a measure for identifying low language abilities in children aged 24–36 months
Source: BMC Pediatr. 2023 Sep 29;23:495. doi: 10.1186/s12887-023-04079-x (PMC10540411; doi:10.1186/s12887-023-04079-x)
Supplement: Supplementary file 3 — Supplementary Material 3 [file 12887_2023_4079_MOESM3_ESM.docx]

Additional file 3: Productivity figures for monolingual English families

|  | **Sensitivity** | **Specificity** | **PLR** | **NLR** | **PPV** | **NPV** |
| --- | --- | --- | --- | --- | --- | --- |
| Section 1 Developmental milestones | 0·98 | 0·56 | 2·23 | 0·04 | 0·20 | >0·99 |
| Section 2 Word list | 0·85 | 0·84 | 5·43 | 0·18 | 0·38 | 0·98 |
| Section 3 Population risk factors | 0·78 | 0·57 | 1·82 | 0·38 | 0·17 | 0·96 |
| Section 4 Professional observations | 0·93 | 0·67 | 2·86 | 0·10 | 0·24 | 0·99 |
| Section 5 Parental concerns | 0·85 | 0·75 | 3·34 | 0·20 | 0·27 | 0·98 |
|  |  |  |  |  |  |  |
| Section 1 & 2 | 0·98 | 0·55 | 2·18 | 0·04 | 0·19 | >0·99 |
| Section 1 & 3 | 0·98 | 0·34 | 1·47 | 0·06 | 0·14 | 0·99 |
| Section 1 & 4 | 0·98 | 0·45 | 1·79 | 0·05 | 0·17 | 0·99 |
| Section 1 & 5 | 0·98 | 0·51 | 1·99 | 0·04 | 0·18 | >0·99 |
| Section 2 & 3 | 0·98 | 0·50 | 1·95 | 0·04 | 0·18 | >0·99 |
| **Section 2 & 4** | **0**·**96** | **0**·**64** | **2**·**69** | **0**·**07** | **0**·**23** | **0**·**99** |
| Section 2 & 5 | 0·91 | 0·71 | 3·12 | 0·12 | 0·26 | 0·99 |
| Section 3 & 4 | 0·98 | 0·38 | 1·57 | 0·06 | 0·15 | 0·99 |
| Section 3 & 5 | 0·98 | 0·45 | 1·77 | 0·05 | 0·16 | 0·99 |
| Section 4 & 5 | 0·96 | 0·59 | 2·33 | 0·07 | 0·21 | 0·99 |
|  |  |  |  |  |  |  |
| Section 1, 2 & 3 | 0·98 | 0·33 | 1·46 | 0·07 | 0·14 | 0·99 |
| Section 1,2,& 4 | 0·98 | 0·45 | 1·77 | 0·05 | 0·16 | 0·99 |
| Section 1,2 & 5 | 0·98 | 0·50 | 1·97 | 0·04 | 0·18 | >0·99 |
| Section 1, 3 & 4 | 0·98 | 0·27 | 1·34 | 0·08 | 0·13 | 0·99 |
| Section 1,3 & 5 | 0·98 | 0·31 | 1·41 | 0·07 | 0·14 | 0·99 |
| Section 2,3 & 4 | 0·98 | 0·36 | 1·53 | 0·06 | 0·15 | 0·99 |
| Section 2,3 & 5 | 0·98 | 0·42 | 1·69 | 0·05 | 0·16 | 0·99 |
| Section 2, 4 & 5 | 0·96 | 0·58 | 2·25 | 0·08 | 0·20 | 0·99 |
| Section 3,4 & 5 | 0·98 | 0·35 | 1·51 | 0·06 | 0·14 | 0·99 |
|  |  |  |  |  |  |  |
| Section 1,2,3,& 4 | 0·98 | 0·26 | 1·33 | 0·08 | 0·13 | 0·99 |
| Section 1, 2,3 & 5 | 0·98 | 0·30 | 1·40 | 0·07 | 0·13 | 0·99 |
| Section 1,2,4 & 5 | 0·98 | 0·41 | 1·66 | 0·05 | 0·16 | 0·99 |
| Section 1,3,4 & 5 | 0·98 | 0·25 | 1·30 | 0·09 | 0·13 | 0·99 |
| Section 2,3,4 & 5 | 0·98 | 0·34 | 1·47 | 0·06 | 0·14 | 0·99 |
