# Supplementary material for: The development and productivity of a measure for identifying low language abilities in children aged 24–36 months
Source: BMC Pediatr. 2023 Sep 29;23:495. doi: 10.1186/s12887-023-04079-x (PMC10540411; doi:10.1186/s12887-023-04079-x)
Supplement: Supplementary file 4 — Supplementary Material 4 [file 12887_2023_4079_MOESM4_ESM.docx]

Additional file 4: Productivity figures for Children from families who speak language other than English

|  | **Sensitivity** | **Specificity** | **PLR** | **NLR** | **PPV** | **NPV** |
| --- | --- | --- | --- | --- | --- | --- |
| Section 1 Developmental milestones | 0·71 | 0·58 | 1·70 | 0·49 | 0·16 | 0·95 |
| Section 2 Word list | 0·93 | 0·89 | 8·82 | 0·08 | 0·49 | 0·99 |
| Section 3 Population risk factors | 0·57 | 0·79 | 2·71 | 0·54 | 0·23 | 0·94 |
| Section 4 Professional observations | 0·79 | 0·63 | 2·13 | 0·34 | 0·19 | 0·96 |
| Section 5 Parental concerns | 0·50 | 0·79 | 2·38 | 0·63 | 0·21 | 0·93 |
|  |  |  |  |  |  |  |
| Section 1 & 2 | 0·93 | 0·58 | 2·21 | 0·12 | 0·20 | 0·99 |
| Section 1 & 3 | 0·86 | 0·53 | 1·81 | 0·27 | 0·17 | 0·97 |
| Section 1 & 4 | 0·93 | 0·42 | 1·60 | 0·17 | 0·15 | 0·98 |
| Section 1 & 5 | 0·71 | 0·53 | 1·51 | 0·54 | 0·14 | 0·94 |
| Section 2 & 3 | 0·93 | 0·74 | 3·53 | 0·10 | 0·28 | 0·99 |
| **Section 2 & 4** | **1**·**00** | **0**·**58** | **2**·**38** | **0**·**00** | **0**·**21** | **>0·99** |
| Section 2 & 5 | 0·93 | 0·74 | 3·53 | 0·10 | 0·28 | 0·99 |
| Section 3 & 4 | 0·93 | 0·53 | 1·96 | 0·14 | 0·18 | 0·99 |
| Section 3 & 5 | 0·79 | 0·74 | 2·99 | 0·29 | 0·25 | 0·97 |
| Section 4 & 5 | 0·86 | 0·47 | 1·63 | 0·30 | 0·15 | 0·97 |
|  |  |  |  |  |  |  |
| Section 1, 2 & 3 | 0·93 | 0·53 | 1·96 | 0·14 | 0·18 | 0·99 |
| Section 1,2,& 4 | 1·00 | 0·42 | 1·73 | 0·00 | 0·16 | >0·99 |
| Section 1,2 & 5 | 0·93 | 0·53 | 1·96 | 0·14 | 0·18 | 0·99 |
| Section 1, 3 & 4 | 1·00 | 0·37 | 1·58 | 0·00 | 0·15 | >0·99 |
| Section 1,3 & 5 | 0·86 | 0·53 | 1·81 | 0·27 | 0·17 | 0·97 |
| Section 2,3 & 4 | 1·00 | 0·53 | 2·11 | 0·00 | 0·19 | >0·99 |
| Section 2,3 & 5 | 0·93 | 0·68 | 2·94 | 0·10 | 0·25 | 0·99 |
| Section 2, 4 & 5 | 1·00 | 0·47 | 1·90 | 0·00 | 0·17 | >0·99 |
| Section 3,4 & 5 | 1·00 | 0·47 | 1·90 | 0·00 | 0·17 | >0·99 |
|  |  |  |  |  |  |  |
| Section 1,2,3,& 4 | 1·00 | 0·37 | 1·58 | 0·00 | 0·15 | >0·99 |
| Section 1, 2,3 & 5 | 0·93 | 0·53 | 1·96 | 0·14 | 0·18 | 0·99 |
| Section 1,2,4 & 5 | 1·00 | 0·37 | 1·58 | 0·00 | 0·15 | >0·99 |
| Section 1,3,4 & 5 | 1·00 | 0·37 | 1·58 | 0·00 | 0·15 | >0·99 |
| Section 2,3,4 & 5 | 1·00 | 0·47 | 1·90 | 0·00 | 0·17 | >0·99 |
